# Exploration of the methodological quality and clinical usefulness of a cross-sectional sample of published guidance about exercise training and physical activity for the secondary prevention of coronary heart disease

**DOI:** 10.1186/s12872-017-0589-z

**Published:** 2017-06-13

**Authors:** Bridget Abell, Paul Glasziou, Tammy Hoffmann

**Affiliations:** Centre for Research in Evidence-Based Practice, Faculty of Health Sciences and Medicine, Bond University, QLD, Gold Coast, 4229 Australia

**Keywords:** Guidelines and statements, Exercise, Secondary prevention, Cardiac rehabilitation

## Abstract

**Background:**

Clinicians are encouraged to use guidelines to assist in providing evidence-based secondary prevention to patients with coronary heart disease. However, the expanding number of publications providing guidance about exercise training may confuse cardiac rehabilitation clinicians. We therefore sought to explore the number, scope, publication characteristics, methodological quality, and clinical usefulness of published exercise-based cardiac rehabilitation guidance.

**Methods:**

We included publications recommending physical activity, exercise or cardiac rehabilitation for patients with coronary heart disease. These included systematically developed clinical practice guidelines, as well as other publications intended to support clinician decision making, such as position papers or consensus statements. Publications were obtained via electronic searches of preventive cardiology societies, guideline databases and PubMed, to November 2016. Publication characteristics were extracted, and two independent assessors evaluated quality using the 23-item Appraisal of Guidelines Research and Evaluation II (AGREE) tool.

**Results:**

Fifty-four international publications from 1994 to 2016 were identified. Most were found on preventive cardiology association websites (*n* = 35; 65%) and were freely accessible (*n* = 50; 93%). Thirty (56%) publications contained only broad recommendations for physical activity and cardiac rehabilitation referral, while 24 (44%) contained the necessary detailed exercise training recommendations. Many were labelled as “guidelines”, however publications with other titles (e.g. scientific statements) were common (*n* = 24; 44%). This latter group of publications contained a significantly greater proportion of detailed exercise training recommendations than clinical guidelines (*p* = 0.017). Wide variation in quality also existed, with ‘applicability’ the worst scoring AGREE II domain for clinical guidelines (mean score 53%) and ‘rigour of development’ rated lowest for other guidance types (mean score 33%).

**Conclusions:**

While a large number of guidance documents provide recommendations for exercise-based cardiac rehabilitation, most have limitations in either methodological quality or clinical usefulness. The lack of rigorously developed guidelines which also contain necessary detail about exercise training remains a substantial problem for clinicians.

**Electronic supplementary material:**

The online version of this article (doi:10.1186/s12872-017-0589-z) contains supplementary material, which is available to authorized users.

## Background

Clinical practice guidelines have become a key tool in delivering evidence-based, high quality health care. Ideally, they provide a systematic synthesis of the expansive base of research evidence, and support clinicians with recommendations based on “an assessment of the benefits and harms of alternative care options” [1]^(p4)^. The aim of guidelines is to assist in the achievement of consistent, effective, and appropriate health care for clinical conditions, thereby bridging the gap between research evidence and practice.

The number of guidelines providing recommendations for the use of exercise training and cardiac rehabilitation in the secondary prevention of coronary heart disease has rapidly expanded. These publications are produced by a variety of bodies, including both professional societies and government organisations. A search of the National Guideline Clearinghouse identifies 78 publications related to cardiac rehabilitation [2], and most national preventive cardiology associations also provide their own list of guidelines, many overlapping the same clinical areas. This duplication means clinicians are faced with the significant challenge of sifting through multiple national and international cardiac rehabilitation guidelines and determining, in a timely manner, which are both trustworthy and relevant to their own practice. This task is compounded by a lack of standardisation in guideline format, ongoing updates and revisions of publications, and different scales for grading the strength of evidence underpinning recommendations [3].

Recommendations intended to assist in the delivery of evidence-based practice may additionally be found in other types of guidance documents such as scientific statements and position papers. These types of publications, often produced by professional associations, are useful to clinicians as they are typically designed to provide more detailed information about topics (such as exercise training) which are considered too specific to address in broader disease focused clinical guidelines [4]. Until recently it was common for these documents to also be badged as guidelines [1]. It is now recognised however, that these various publications differ from clinical guidelines in their development processes and incorporation of evidence, which may impact upon methodological quality [1, 4]. Consequently, while these other forms of ‘clinical guidance’ are often of value to clinicians, they should be distinguished from more methodologically rigorous, evidence-based ‘clinical practice guidelines’ [1]. Whether clinicians who are unversed in guideline development and terminology can easily make this distinction is however unclear [5, 6].

Despite the proliferation and widespread availability of both clinical practice guidelines and other guidance documents in cardiac rehabilitation, there is limited research documenting the total number, characteristics, quality, and scope of these publications. A previous examination [7] included only clinical practice guidelines, and was limited by strict inclusion criteria to 9 publications. Many documents which are produced by professional associations, and potentially used by clinicians to guide practice, were not included. The characteristics of different guidelines and guidance documents potentially encountered by cardiac rehabilitation clinicians therefore remain largely unexplored. Consequently, we aimed to explore the scope, publication characteristics, and methodological quality of all publications which recommend the use of exercise, physical activity, or cardiac rehabilitation in the secondary prevention of coronary heart disease. Additionally, we examined how these publication characteristics may be related to guidance document type and usefulness in clinical practice.

## Methods

### Design

Analysis of a cross-sectional sample of publications.

### Inclusion criteria

To be included, publications were required to provide recommendations intended to optimise the use of cardiac rehabilitation, physical activity, or exercise for people with established coronary heart disease (including myocardial infarction, angina, coronary artery bypass graft, and percutaneous coronary intervention). We excluded publications concerned solely with the primary prevention of coronary heart disease. To capture the full spectrum of clinical guidance available to clinicians, we included systematically developed clinical practice guidelines [1], as well as other publications intended to support clinician decision making in cardiac rehabilitation, such as scientific statements, position stands, or consensus statements. Publications which provided reviews of the evidence without recommendations for application in practice were excluded. Only guidance in English was included, with no limit on the date of publication. Where more than one version of a guideline or publication was identified, only the most recent update was included. However, older versions were searched for information regarding the development process if required.

### Search strategy

Initially we searched for appropriate references within the web-based resources and publications of cardiology societies and cardiac rehabilitation associations in Australia, New Zealand, the United Kingdom, the United States, Canada, and Europe (see Additional file 1 a for full list). We also searched in databases and organisations known to either compile or develop international guidelines (full list in Additional file 1 b). The strategy most appropriate for each database was used, and generally consisted of a search using the terms “cardiac rehabilitation”, “exercise”, and/or “secondary prevention”. If the number of potentially relevant publications listed within the database was less than 100, a manual search of all records filed under the following categories (where they existed) was conducted: “secondary prevention”, “coronary artery disease”, “cardiovascular disease”, “cardiology”, “myocardial infarction”, “percutaneous coronary intervention”, “coronary artery bypass graft”, “exercise”, “physical activity”, “risk factors”, “lifestyle modification”, or “cardiac rehabilitation”. Finally, we conducted a search of the PubMed, PEDro, and TRIP databases using the text term “cardiac rehabilitation” and limiting the publication type to guidelines, practice guidelines and consensus development conferences. Searches were conducted in November 2016.

### Data collection and classification

Characteristics of included publications (e.g. year of release, publishing body) were collected using a standardised Excel spreadsheet. We additionally used the title, abstract, preamble, and introduction to classify publications according to three different scopes of information provided: (a) the general diagnosis and management of cardiovascular conditions or cardiovascular risk; (b) guidance about cardiac rehabilitation interventions; or (c) guidance about exercise or physical activity for cardiovascular disease. Publications were also classified based on the type of recommendations provided, either: (a) broad recommendations about the need for cardiac rehabilitation referral and/or general physical activity advice; or (b) detailed and specific recommendations for exercise training or protocols during cardiac rehabilitation. Finally, we classified publications according to guidance document type (e.g. clinical guidelines, position stands, scientific statements), based on their self-reported title.

### Appraisal of clinical guidelines and guidance documents

The scientific and methodological quality of each included publication was independently evaluated by two researchers using the Appraisal of Guidelines Research and Evaluation II (AGREE II) instrument [8]. Both reviewers completed the online AGREE II training module [9] before commencing appraisal. The AGREE II instrument contains 23 items, arranged into 6 independent domains: 1) reported scope and purpose; 2) stakeholder involvement; 3) rigour of development, including evidence and formation of recommendations; 4) clarity of presentation; 5) applicability and implementation; and 6) editorial independence (Additional file 2). A final item additionally rates overall quality of the guideline. The AGREE II instrument has been used previously by others to evaluate various types of consensus statements [10, 11].

The online My AGREE PLUS platform [12] and associated manual was used by both researchers for data collection, judgement of item ratings, and overall domain scoring. Each item was rated on a 7-point Likert scale from 1 (strongly disagree or no information reported about item) to 7 (strongly agree that reporting of the item meets full criteria in AGREE II user manual). Discrepancies in item rating between assessors differing by more than 2 points were discussed, with an opportunity to individually re-rate these items if deemed appropriate. When rating each AGREE item, information from the publication as a whole was considered, however particular emphasis was placed on the sections describing exercise, physical activity or cardiac rehabilitation when rating the domains for rigour of development, clarity of presentation, and applicability and implementation. The score for each domain was calculated as a percentage of the maximum score possible across all items in that domain (e.g. a domain with 3 items and 2 reviewers would have a maximum score of 42).

### Data analysis

Descriptive statistics (range, frequency, percentage) were used to analyse the characteristics and scope of included publications. Chi-squared tests were used to examine the hypotheses that there were differences in the main scope and clinical usefulness (detailed vs broad exercise recommendations) of publications based on guidance document classification (clinical guideline vs other). A Bonferroni adjusted alpha level of 0.025 was used due to multiple comparisons. All analyses were performed using SPSS Statistics 23.0 (SPSS Inc. Chicago IL).

## Results

### Selection of publications

**Fig. 1:**
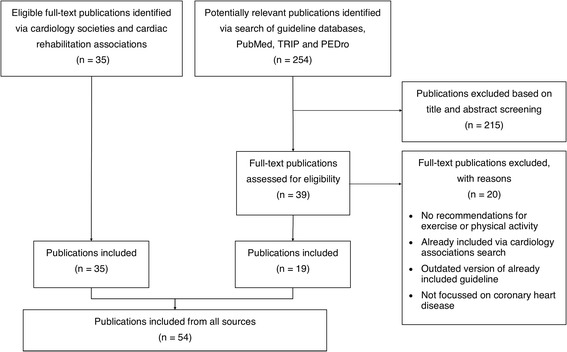
Publication searching, screening and selection process

Figure 1 shows the results of the searching and screening process for guidance selection. A total of 54 publications were eligible for inclusion in our analysis. A detailed list of these documents is provided in Additional file 3.

### Characteristics of publications

The included publications were dated between 1994 and 2016, with almost two-thirds (61%) published within the previous 5 years, and most as updates to previous guidance (69%) (Table 1). Publications from the United States (37%) and Europe (22%) accounted for over half of all guidance. The 54 documents included journal articles, stand-alone reports, and hardcopy books. The three books were published by professional associations, and all required the user to purchase a copy to obtain access. Additionally, one journal article was only accessible via subscription. Journal-based guidance was most often found in the official publication of the professional association responsible for its release. Eleven different journals were represented (Additional file 3) and all were indexed in PubMed.

**Table 1:** Characteristics of 54 publications providing guidance about exercise in coronary heart disease

Characteristic or classification	Number of publications (%)
Year of publication	
1990–1995	1 (1.9)
1996–2000	3 (5.6)
2001–2005	9 (16.7)
2006–2010	8 (14.8)
2011–2015	31 (57.4)
2016-present	2 (3.7)
Region represented by publishing organisation	
United States of America	20 (37.0)
Europe	12 (22.2)
United Kingdom	9 (16.7)
Australia	7 (13.0)
Canada	4 (7.4)
New Zealand	2 (3.7)
Type of publication	
Journal article	32 (59.3)
Standalone report	18 (33.3)
Book	3 (5.6)
Other (found in both a journal and as a report)	1 (1.9)
Indexed by PubMed	
Yes	38 (70.4)
Access free of charge	
Yes	50 (92.6)
Update of previous publication	
Yes	37 (68.6)

### Classification of publications

#### By scope of publication

Just over half (52%) of the included publications focussed on the general diagnosis and management of a range of cardiovascular conditions such as acute coronary syndromes, percutaneous coronary intervention, or coronary artery bypass grafting (Table 2 and Additional file 3). The remainder focussed specifically on the use of cardiac rehabilitation interventions (28%), or exercise therapy and physical activity (20%), for patients with coronary heart disease.

**Table 2:** Classification of 54 publications providing guidance about exercise in coronary heart disease

Characteristic or classification	Number of publications (%)
Main scope of publication	
General diagnosis/management of various cardiovascular conditions	28 (51.9)
To provide cardiac rehabilitation (CR) guidance	15 (27.8)
To provide exercise or physical activity guidance	11 (20.4)
Type of exercise recommendation	
Detailed: Specific recommendations for exercise protocol or CR	24 (44.4)
Broad: Physical activity recommendations and/or advocate referral to CR	30 (55.6)
*Physical activity recommendations and advocate referral to CR*	*23*
*Advocate referral to CR*	*5*
*Provide physical activity recommendations only*	*2*
Classification of guidance type	
Clinical Guideline	30 (55.6)
Scientific Statement	7 (13.0)
Position Stands/Statement	7 (13.0)
Consensus Statement	3 (5.6)
Core Components	2 (3.7)
Guides	2 (3.7)
Recommendations	2 (3.7)
Standards	1 (1.9)

#### By type of exercise recommendation (clinical usefulness)

Of the 30 publications offering broad recommendations, 23 (77%) advocated referral to formal cardiac rehabilitation programs for most patients, but did not offer further guidance about the format these programs should take (Table 2). Instead, they made general recommendations for increasing physical activity levels as part of a comprehensive risk reduction strategy. A further 5 publications provided the recommendation for routine cardiac rehabilitation referral but did not provide any additional physical activity advice, while another 2 offered physical activity advice without explicitly recommending referral to formal cardiac rehabilitation programs.

Detailed recommendations for the exercise training or protocols to be used during cardiac rehabilitation were provided in 24 publications. Nineteen (79%) of these were found within the resources and publication sections of national cardiac rehabilitation associations or cardiology societies. Seven of these documents could only be found via these sources, and were not indexed in PubMed or listed in guideline databases.

#### By type of guidance document

The majority of guidance documents (56%) described themselves as clinical guidelines. Exercise and physical activity recommendations were also found however in other publications with titles such as scientific statements (13%) and position papers (13%) (Table 2).

A comparison of the clinical usefulness and main scope of these different types of document is presented in Table 3. A significant relationship was observed between the type of guidance document and the main scope of each publication (*p* = 0.0004). Cardiovascular disease management and diagnosis were more likely to be the main scope of publications classified as clinical guidelines (22/30; 73%), while other types of guidance publication had a greater focus on topics such as exercise, physical activity, and cardiac rehabilitation (18/24; 75%). Additionally, the proportion of publications which contained detailed exercise training recommendations (rather than broad referral and physical activity advice) varied significantly by the type of guidance document, with clinical guidelines less likely to contain more detailed recommendations compared to other guidance types (*p* = 0.017). Just 30% of the publications classified as clinical guidelines contained these detailed recommendations compared to almost two-thirds of other guidance types (15/24; 63%).

**Table 3:** Clinical guidelines vs. other guidance types: comparison of publication scope and type of exercise recommendations

Type of guidance document	Main publication focus/scope			Type of exercise recommendation	
	*General disease management/diagnosis*	*Cardiac rehabilitation*	*Exercise/physical activity*	*Broad*	*Detailed*
Clinical guideline (*n* = 30)	22 (73.3%)	6 (20%)	2 (6.7%)	21 (70%)	9 (30%)
Other guidance document e.g. scientific statement (*n* = 24)	6 (25%)	9 (37.5%)	9 (37.5%)	9 (37.5%)	15 (62.5%)

### Quality of publications

Standardised domain scores from the AGREE II assessment are displayed for each included guidance publication in Additional file 4.

#### Clinical practice guidelines

Domain scores from the AGREE II assessment of clinical practice guidelines are displayed in Table 4. Across all 30 publications, the ‘applicability’ domain (reporting on implementation strategies, barriers, resources, and cost) had the lowest mean score (53%). The highest scoring domain was ‘clarity of presentation’ (language, structure, unambiguous and identifiable recommendations), with a mean rating of 79%. Ratings for the ‘editorial independence’ domain showed the largest variation, with one publication scoring 0% in this domain (missing or poor statements dealing with funding bodies and conflicts of interest) and one scoring 100%. The National Institute for Health and Care Excellence guidelines, UK1 [13], UK2 [14], UK4 [15], and UK5 [16] consistently scored high domain ratings (Additional file 4).

**Table 4:** AGREE II domain scores across all publications classified as clinical guidelines (*n* = 30)

AGREE II Domain (%)	Minimum	Maximum	Median	Mean	Standard deviation	Lowest rated^a^	Highest rated^a^
Scope and purpose	25	100	68	68	19	USA11	UK1
Stakeholder involvement	19	92	52	56	19	EUR11	UK2
Rigour of development	32	92	68	65	16	EUR11	UK1
Clarity of presentation	58	97	78	79	10	USA18	CAN1, USA2
Applicability	10	79	56	53	21	CAN3	UK1
Editorial Independence	0	100	88	74	28	USA5	USA20

^a^I.D code for publications as listed in Additional file 3 with full reference and description

#### Other forms of guidance

Table 5 presents the AGREE II domain scores for all other types of guidance publication (e.g. consensus statement, position paper). Within this group of publications, the ‘rigour of development’ domain (systematic search and appraisal of evidence, clear formulation of recommendations, links to evidence used) displayed the lowest mean score (30%), with the highest rated publication [17] scoring only 55%. As with clinical practice guidelines, ‘clarity of presentation’ was the highest scoring domain and ‘editorial independence’ had the widest range of scores.

**Table 5:** AGREE II domain scores across publications classified as other types of guidance documents (*n* = 24)

AGREE II Domain (%)	Minimum	Maximum	Median	Mean	Standard deviation	Lowest rated^a^	Highest rated^a^
Scope and purpose	44	86	63	63	10	EUR6	UK6
Stakeholder involvement	8	64	33	36	14	USA14	UK3, AUS2
Rigour of development	12	55	28	30	11	EUR4	AUS2
Clarity of presentation	42	92	69	69	12	USA16	EUR5
Applicability	10	75	31	33	16	EUR12	EUR4
Editorial Independence	0	96	63	50	35	EUR7, AUS6, EUR8	EUR4

^a^I.D code for publications as listed in Additional file 3 with full reference and description

## Discussion

This study systematically explored the scope, characteristics, and methodological quality of a range of publications which provide guidance for exercise-based cardiac rehabilitation and physical activity in coronary heart disease. We identified 54 guidance publications which were produced by numerous national and international associations, many overlapping in clinical focus. Not all of these were published in journals, nor titled as clinical guidelines, nor indexed in PubMed, but most were freely available and recently produced. Methodological quality was generally modest, and varied within and between clinical guidelines and other types of guidance. The domains of ‘applicability’, ‘stakeholder involvement', and ‘rigour of development’ were particularly poor across most publications.

While the methodological quality of guidelines in many areas of healthcare has been assessed, there is limited research exploring the publication characteristics of these documents. Studies which have appraised guideline quality in other clinical areas have observed similar findings to those in our sample, in that a large proportion of guidance is produced in the United States [18, 19], is obtained from professional organisations [10, 20], and often comprises publication types with titles other than clinical guidelines [10, 11, 21].

A recent study [7] which appraised the quality of a number of cardiac rehabilitation guidelines, did so for only nine publications (7 of which were included in our sample) due to strict inclusion criteria and a failure to search the resources of professional associations. Of these publications, only four provided detailed exercise protocols, and one has since been archived due to its age. Additionally, none of the included guidelines had been published within the last 5 years. Our study used broader criteria and included all publications which may be currently encountered by clinicians in their search for guidance, thereby enabling better informed choices about which types of guidance to use to aid in the decision-making process. Nevertheless, in both studies guidelines scored lowest in the ‘applicability’ domain, and highest for ‘clarity of presentation’.

Our finding that the domain of ‘applicability’ scored poorly in exercise and cardiac rehabilitation guidance is not unique to this area. ‘Applicability’ has been identified as the lowest rated domain in guidelines for cancer-related fatigue [22], biologic agents [11] or physiotherapy [23] in rheumatoid arthritis, hyponatremia [10], genetic screening for colorectal cancer [18], Cushing’s syndrome [24], and breast cancer rehabilitation [25]. This domain is primarily concerned with issues surrounding the implementation of the guideline and should assess and discuss any facilitators, barriers and resourcing implications of applying the recommendations in practice [8]. Additionally, the publication should provide tools and resources to aid clinical implementation (e.g. a brief summary, algorithm, flow chart), as well as clear monitoring criteria for clinicians to assess how well recommendations are being implemented. Guidelines are intended to aid clinicians by not only providing them with ready access to the best available knowledge, but also a means of translating this knowledge into their everyday practice. Although many factors may be responsible for poor uptake of a guideline in practice, research suggests that if implementation tools or strategies can be incorporated within the guideline, some of these issues can be overcome [19]. The low domain scores in this area for all types of cardiac rehabilitation guidance may therefore be hindering the implementation of these recommendations in practice. Given the substantial challenges to health care funding and resourcing cardiac rehabilitation services are currently facing, it is particularly important that resourcing issues, facilitators, and barriers of exercise programs are considered within this guidance. Without access to this knowledge clinicians may be unsure, or incorrectly judge, the impact of implementing recommendations in their own practice.

It is important to also recognised that AGREE scores have not been shown to consistently relate to the uptake of a guideline in practice [19]. While this issue has received little exploration, one possible explanation may be highlighted in our findings, with the higher rated guideline publications less likely to contain recommendations specific enough to translate into care in practice. This is in agreement with guidance for the use of TNFα antagonists in rheumatoid arthritis [11], which demonstrated an increased proportion of detailed treatment and prescribing recommendations in consensus statements, as opposed to clinical guidelines.

The modest AGREE domain scores observed for both clinical guidelines and other guidance publications in this sample may not solely be due to inadequacies in the development processes. Incomplete reporting in many of the publications also contributed to modest scores, as items with missing information could not be rated, whether or not the process actually occurred during development. While this raises the need for better attention to reporting in the future, this poor reporting is not unique to guidelines, but rather is an ongoing problem in many areas of research, including trial methodology [26, 27] and intervention descriptions [28]. Additionally, the inclusion of some older publications may have also contributed towards lower domain scores as guideline quality has generally improved over time [29].

### Implications for practice

A range of potential barriers to guideline implementation in practice have been previously identified and issues with the guideline publications themselves are frequently cited [30, 31]. Across a variety of clinical fields, factors such as the volume of information, clarity and complexity of recommendations, recentness of publication, perceived applicability to practice, and conflicting or confusing publications are repeatedly highlighted as issues. We have demonstrated that this may also be the case in the field of cardiac rehabilitation, with clinicians faced with an overwhelming amount of guidance to decipher. For this reason, many will often rely on the judgement of their own professional organisation for direction in this area [6]. Fortunately, we did find a large proportion of publications available from these sources, and these were more likely to contain the specific exercise and cardiac rehabilitation protocols most useful in practice. However, many of these publications were not titled as guidelines, and lacked the methodological rigour of those which were. Clinicians may be unaware of this fact, and erroneously assume scientific statements and position papers have been subject to the same rigorous development process and appraisal of evidence as guidelines. This is not an issue restricted to cardiac rehabilitation, with differences in methodological quality also observed between clinical guidelines and other types of publications within oncology [21] and rheumatoid arthritis [11] guidance.

#### Clinical guidelines versus other types of guidance publication

While clinical practice guidelines and other types of guidance aim to provide recommendations to optimise patient care, the observed differences in methodological quality between these groups of publications is not unexpected, given their varying aims and development processes. Unlike guidelines, other forms of guidance do not necessarily use a systematic review and grading of the relevant evidence. Instead they are more likely to provide the collective opinion of an expert multidisciplinary panel concerning evidence-based approaches to advancing patient care in particular areas [4, 32, 33]. Additionally, while many professional associations provide methodology manuals for the transparent and systematic development of clinical guidelines [32, 34], such publications for other types of guidance are generally lacking. Consequently, lower and more variable AGREE II scores could be expected in this later group of publications, particularly for ‘rigor of development’.

Furthermore, clinical guidelines are generally designed to provide recommendations where there is a strong evidence base, while guidance such as consensus statements may be developed to inform clinical practice where the evidence is less clear or still evolving [4]. For example, the broad recommendation for referral to exercise-based cardiac rehabilitation that was noted in a large number of guidelines stems from the well documented benefits of this intervention in randomised control trials and systematic reviews [35]. However, evidence for the specific and detailed exercise criteria to prescribe in practice (such as the frequency, intensity and volume of exercise) is less well defined, continually evolving, and may be more reliant on consensus opinion. It is unsurprising therefore that these types of detailed recommendations were more commonly found in types of guidance other than clinical guidelines in our sample of publications.

Finally, the notion that clinical guidelines act as a concise and evidence-based “what to do” rather than “how to do” appears to be consistent with the findings of this study. While these publications do provide broad evidence-based recommendations for exercise-based cardiac rehabilitation, clinicians requiring specific guidance about the “how to” of translating exercise training interventions into practice, would most likely need to source this information from other types of guidance document. The role of publications such as scientific statements in providing the “how to” of evidence translation has previously been acknowledged [1, 4]. Given their potentially greater clinical usefulness, the large gap in methodological quality we observed between these types publications and clinical guidelines is disparaging. Ideally, the development of all types of guidance publications should be transparent and the evidence-base and its interpretation clearly defined to allow assessment of any areas where potential bias may impact the validity of the recommendations.

Given these findings, clinicians should be cautious when searching for and implementing recommendations for exercise-based cardiac rehabilitation in practice. Potential users need to understand the inherent differences between these publication types in order to make judgements about the potential risk of bias, and the necessary trade-off often required between methodological quality and clinical usefulness. The summary of guidance documents provided in this article may assist clinicians in selecting the most trustworthy and relevant guidance publication for their practice, or at least provide an aid in assessing methodological quality. Where it exists, a clinical guideline should be considered above other types of guidance, however this may often not be possible as currently clinicians have access to very few recently developed, rigorous guidelines which also provide the specific and detailed recommendations necessary to implement exercise and cardiac rehabilitation. Relevant journals and professional associations are therefore encouraged to use the AGREE II tool and IOM criteria when developing or updating clinical guidelines. Other types of publications, such as scientific statements, should at least provide clear reporting of the methodology employed and links to the evidence to promote increased transparency. Additionally, the relationships between publications from the same organisation need to be made clearer, as we often had difficulty determining which had overlapping content or had been replaced by newer versions.

### Strengths and limitations

A strength of our study is the use of a comprehensive search strategy with broad selection criteria. This enabled us to identify publications which are representative of all English-language guidance currently available to clinicians working in the field of exercise-based cardiac rehabilitation. We also used a validated tool (AGREE II) to systematically assess and compare the quality and transparency of these publications. By using a standardised checklist for all guidance publications, we were able to assess whether biases in development had been adequately addressed. While the AGREE tool is not specifically designed for assessing guidance documents such as scientific statements, and it could therefore be argued that these non-guideline publications would naturally have lower scores, no other suitable appraisal tool currently exists [11]. As these types of guidance are commonly encountered by clinicians, and are often intended to be used similarly to clinical guidelines, it is important that a summary of their quality exists.

Finally, in analysis we chose to classify publications as clinical guidelines based on their title rather than the established IOM criteria. Consequently, two publications (USA5, USA2) were classified as clinical guidelines in our study, even though they failed to explicitly report a systematic search or assessment of the evidence used to formulated recommendations. However, we felt clinicians who may not be aware of the intricacies of the guideline definition may make the same classification, given that they are titled guidelines and are currently two of the most widely used cardiac rehabilitation resources, particularly in North America.

## Conclusions

We have shown that while an expansive volume of exercise-based cardiac rehabilitation guidance exists, much of it has limitations (in either rigour or usefulness) which may hinder integration of evidence into cardiac rehabilitation practice. While publications are often easily located via professional associations or guideline developers, clinicians need to be aware that these may vary substantially in terms of quality, guidance type, and usefulness of recommendations. There is room for improving the quality of all these publications, and in particular for providing transparent, rigorously developed, evidence-based clinical guidelines with a specific focus on implementable exercise prescriptions for people with coronary heart disease.

## Additional files

Additional file 1: Sources searched for publications. **a** Cardiac Rehabilitation and Cardiology associations searched. **b** Guideline databases searched. (DOCX 15 kb)

Additional file 2: Table. The 6 AGREE II domains and corresponding 23 items. (DOCX 12 kb)

Additional file 3: Table. Details of all included publications. (DOCX 68 kb)

Additional file 4: Table. AGREE II domain scores (%) for all included publications. (DOCX 27 kb)

## Abbreviation

CR: Cardiac rehabilitation
