# Supplementary material for: Exploration of the methodological quality and clinical usefulness of a cross-sectional sample of published guidance about exercise training and physical activity for the secondary prevention of coronary heart disease
Source: BMC Cardiovasc Disord. 2017 Jun 13;17:153. doi: 10.1186/s12872-017-0589-z (PMC5470313; doi:10.1186/s12872-017-0589-z)
Supplement: Supplementary file 1 — Sources searched for publications. a Cardiac Rehabilitation and Cardiology associations searched. b Guideline databases searched. (DOCX 15 kb) [file 12872_2017_589_MOESM1_ESM.docx]

**Additional file 1.** Sources searched for publications

**a.** Cardiac Rehabilitation and Cardiology associations searched

**Australia and New Zealand**

Australian Cardiovascular Health and Rehabilitation Association (ACRA)

Cardiac Society of Australia and New Zealand (CSANZ)

National Heart Foundation Australia (NHFA)

National Heart Foundation New Zealand (NHFNZ)

**United Kingdom**

British Association for Cardiovascular Prevention and Rehabilitation (BACPR)

British Cardiovascular Society (BCS)

Association of Chartered Physiotherapists in Cardiac Rehabilitation (ACPICR)

**Europe**

European Association for Cardiovascular Prevention and Rehabilitation (EACPR)

European Society of Cardiology (ESC)

**United States**

American Association of Cardiovascular and Pulmonary Rehabilitation (AACVPR)

American Heart Association (AHA)

American College of Sports Medicine (ACSM)

**Canada**

Canadian Association of Cardiovascular Prevention and Rehabilitation (CACPR)

Canadian Cardiovascular Society (CCS)

**b**. Guideline databases searched

National Guideline Clearinghouse

Agency for Healthcare Research and Quality

Guideline International Network

National Library of Guidelines (including the National Institute for Clinical Excellence)

Scottish Intercollegiate Guidelines Network

New Zealand Guidelines Group

Canadian Medical Association Infobase

National Health and Medical Research Council
