# Supplementary material for: Exploration of the methodological quality and clinical usefulness of a cross-sectional sample of published guidance about exercise training and physical activity for the secondary prevention of coronary heart disease
Source: BMC Cardiovasc Disord. 2017 Jun 13;17:153. doi: 10.1186/s12872-017-0589-z (PMC5470313; doi:10.1186/s12872-017-0589-z)
Supplement: Supplementary file 2 — Table. The 6 AGREE II domains and corresponding 23 items. (DOCX 12 kb) [file 12872_2017_589_MOESM2_ESM.docx]

**Additional file 2.** The 6 AGREE II domains and corresponding 23 items

| **DOMAIN** | **ITEM AND DESCRIPTION** |
| --- | --- |
| Scope and Purpose | 1. The overall objective(s) of the guideline is (are) specifically described  2. The health question(s) covered by the guideline is (are) specifically described  3. The population (patients, public, etc.) to whom the guideline is meant to apply is specifically described |
| Stakeholder Involvement | 4. The guideline development group includes individuals from all relevant professional groups  5. The views and preferences of the target population (patients, public, etc.) have been sought  6. The target users of the guideline are clearly defined |
| Rigour of Development | 7. Systematic methods were used to search for evidence  8. The criteria for selecting the evidence are clearly described  9. The strengths and limitations of the body of evidence are clearly described  10. The methods for formulating the recommendations are clearly described  11. The health benefits, side effects, and risks have been considered in formulating the recommendations  12. There is an explicit link between the recommendations and the supporting evidence  13. The guideline has been externally reviewed by experts prior to its publication  14. A procedure for updating the guideline is provided |
| Clarity of Presentation | 15. The recommendations are specific and unambiguous  16. The different options for management of the condition or health issue are clearly presented  17. Key recommendations are easily identifiable |
| Applicability | 18. The guideline describes facilitators and barriers to its application  19. The guideline provides advice and/or tools on how the recommendations can be put into practice  20. The potential resource implications of applying the recommendations have been considered  21. The guideline presents monitoring and/or auditing criteria |
| Editorial Independence | 22. The views of the funding body have not influenced the content of the guideline  23. Competing interests of guideline development group members have been recorded and addressed |

**From:** Brouwers MC, Kho ME, Browman GP, Burgers JS, Cluzeau F, Feder G, Fervers B, Graham ID, Grimshaw J, Hanna SE, Littlejohns P, Makarski J, Zitzelsberger L. AGREE II: advancing guideline development, reporting and evaluation in health care. CMAJ: Canadian Medical Association Journal, 2010;182:E839-E842
