# Supplementary material for: Exploration of the methodological quality and clinical usefulness of a cross-sectional sample of published guidance about exercise training and physical activity for the secondary prevention of coronary heart disease
Source: BMC Cardiovasc Disord. 2017 Jun 13;17:153. doi: 10.1186/s12872-017-0589-z (PMC5470313; doi:10.1186/s12872-017-0589-z)
Supplement: Supplementary file 3 — Table. Details of all included publications. (DOCX 68 kb) [file 12872_2017_589_MOESM3_ESM.docx]

**fAdditional file 3.** Details of all included publications

| **I.D** | **Publication Title (reference)** | **Year** | **Region** | **Publication Type** | **Classification of Guidance Type** | **Type of Exercise Recommendation** | **Update** | **Publisher** | **Journal** | **Free Access** | **Where Found** | **Indexed in PubMed** | **Main Content Focus** |
| --- | --- | --- | --- | --- | --- | --- | --- | --- | --- | --- | --- | --- | --- |
| USA1 | Secondary prevention of ischemic heart disease and stroke in adults [1] | 2014 | USA | Report | CGL | PA + RCR | Yes | University of Michigan Health System | n/a | Yes | NGC | No | General |
| USA2 | ACSM's guidelines for exercise testing and prescription (9th edition). Chapter 9: Exercise prescription for patients with cardiovascular and cerebrovascular disease [2] | 2014 | USA | Book | CGL | CRS | Yes | American College of Sports Medicine | n/a | No | AACVPR | No | Exercise |
| USA3 | Secondary prevention of atherosclerotic cardiovascular disease in older adults [3] | 2013 | USA | Journal | SS | PA + RCR | Yes | American Heart Association | Circulation | Yes | AHA | Yes | General |
| USA4 | Stable coronary artery disease [4] | 2013 | USA | Report | CGL | PA + RCR | Yes | Institute for Clinical Systems Improvement | n/a | Yes | NGC | No | General |
| USA5 | Guidelines for cardiac rehabilitation and secondary prevention programs, 5th edition [5] | 2013 | USA | Book | CGL | CRS | Yes | American Association of Cardiovascular and Pulmonary Rehabilitation | n/a | No | AACVPR | No | Cardiac Rehabilitation |
| USA6 | Guideline for the diagnosis and management of patients with stable ischemic heart disease [6,7] | 2012/2014 | USA | Journal | CGL | PA + RCR | Yes | ACCF/AHA/ACP/AATS/PCNA/SCAI/STS | Journal of the American College of Cardiology; Circulation | Yes | NGC | Yes | General |
| USA7 | AHA/ACCF Secondary prevention and risk reduction therapy for patients with coronary and other atherosclerotic vascular disease: 2011 update [8] | 2011 | USA | Journal | CGL | PA + RCR | Yes | American Heart Association; American College of Cardiology | Circulation | Yes | AHA | Yes | General |
| USA8 | Diagnosis and treatment of chest pain and acute coronary syndrome [9] | 2012 | USA | Report | CGL | CRS | Yes | Institute for Clinical Systems Improvement | n/a | Yes | NGC | No | General |
| USA9 | Guideline for percutaneous coronary intervention [10,11] | 2011/2015 | USA | Journal | CGL | RCR | Yes | AHA/ACCF/SCAI | Journal of the American College of Cardiology; Circulation | Yes | NGC | Yes | General |
| USA10 | Guideline for the management of ST-elevation myocardial infarction [11,12] | 2013/2015 | USA | Journal | CGL | RCR | Yes | American Heart Association; American College of Cardiology | Journal of the American College of Cardiology; Circulation | Yes | NGC | Yes | General |
| USA11 | 2007 Chronic angina focused update of the 2002 guidelines for the management of patients with chronic stable angina [13] | 2007 | USA | Journal | CGL | PA + RCR | Yes | American Heart Association; American College of Cardiology | Circulation | Yes | AHA | Yes | General |
| USA12 | Core components of cardiac rehabilitation/secondary prevention programs: 2007 update [14] | 2007 | USA | Journal | SS | CRS | Yes | American Heart Association; American Association of Cardiovascular and Pulmonary Rehabilitation | Circulation | Yes | AHA | Yes | Cardiac Rehabilitation |
| USA13 | Exercise standards for testing and training [15] | 2013 | USA | Journal | SS | CRS | Yes | American Heart Association | Circulation | Yes | AHA/ AACVPR | Yes | Exercise |
| USA14 | Exercise for patients with coronary artery disease [16] | 1994 | USA | Journal | PS | CRS | No | American College of Sports Medicine | Medicine and Science in Sports and Exercise | Yes | ACSM | Yes | Exercise |
| USA15 | Resistance exercise in individuals with and without cardiovascular disease: 2007 update [17] | 2007 | USA | Journal | SS | CRS | Yes | American Heart Association | Circulation | Yes | AHA | Yes | Exercise |
| USA16 | Cardiac rehabilitation and secondary prevention of coronary heart disease [18] | 2005 | USA | Journal | SS | RCR | Yes | American Heart Association | Circulation | Yes | AHA | Yes | Cardiac Rehabilitation |
| USA17 | Exercise and physical activity in the prevention and treatment of atherosclerotic cardiovascular disease [19] | 2003 | USA | Journal | SS | PA + RCR | No | American Heart Association | Circulation | Yes | AHA | Yes | Exercise |
| USA18 | Effectiveness-based guidelines for the prevention of cardiovascular disease in women—2011 update [20] | 2011 | USA | Journal | CGL | PA + RCR | Yes | American Heart Association | Circulation | Yes | NCG | Yes | General |
| USA19 | Secondary prevention after coronary artery bypass graft surgery [21] | 2015 | USA | Journal | SS | RCR | Yes | American Heart Association | Circulation | Yes | AHA | Yes | General |
| USA20 | 2014 AHA/ACC guideline for the management of patients with non–ST-elevation acute coronary syndromes [22] | 2014 | USA | Journal | CGL | PA + RCR | Yes | American Heart Association | Circulation | Yes | AHA | Yes | General |
| CAN1 | Canadian guidelines for cardiac rehabilitation and cardiovascular disease prevention: Translating knowledge into action, 3rd edition [23] | 2009 | CAN | Book | CGL | CRS | Yes | Canadian Association of Cardiovascular Prevention and Rehabilitation | n/a | No | CACPR | Yes* | Cardiac Rehabilitation |
| CAN2 | Canadian Cardiovascular Society 2000 consensus conference: Women and ischemic heart disease [24] | 2000 | CAN | Journal | CS | PA + RCR | No | Canadian Cardiovascular Society | Canadian Journal of Cardiology | Yes | CCVS/ CMA Infobase | Yes | General |
| CAN3 | Canadian Cardiovascular Society guidelines for the diagnosis and management of stable ischemic heart disease [25] | 2014 | CAN | Journal | CGL | PA + RCR | Yes | Canadian Cardiovascular Society | Canadian Journal of Cardiology | Yes | CCVS/ CMA Infobase | Yes | General |
| CAN4 | Management of heart disease in the elderly patient [26] | 2002 | CAN | Report | CS | CRS | No | Canadian Cardiovascular Society | n/a | Yes | CCVS/ CMA Infobase | Yes | General |
| UK1 | Cardiovascular disease: risk assessment and reduction, including lipid modification, #181 | 2014 | UK | Report | CGL | PAA | Yes | National Institute for Health and Care Excellence | n/a | Yes | NICE | No | General |
| UK2 | MI – secondary prevention: Secondary prevention in primary and secondary care for patients following a myocardial infarction, #172 [27] | 2013 | UK | Report | CGL | PA + RCR | Yes | National Institute for Health and Care Excellence | n/a | Yes | NICE | Yes | General |
| UK3 | BACPR standards and core components for cardiovascular disease prevention and rehabilitation 2012 [28] | 2012 | UK | Report | CC | CRS | Yes | British Association for Cardiovascular Prevention and Rehabilitation | n/a | Yes | BACPR | No | Cardiac Rehabilitation |
| UK4 | Management of stable angina, #126 [29] | 2012 | UK | Report | CGL | PAA | Yes | National Institute for Health and Care Excellence | n/a | Yes | NICE | Yes* | General |
| UK5 | Unstable angina and NSTEMI: The early management of unstable angina and non-ST-segment-elevation myocardial infarction, #94 [30] | 2013 | UK | Report | CGL | PA + RCR | Yes | National Institute for Health and Care Excellence | n/a | Yes | NICE | Yes | General |
| UK6 | ACPICR standards for physical activity and exercise in the cardiac population 2015 [31] | 2015 | UK | Report | ST | CRS | Yes | Association of Chartered Physiotherapists in Cardiac Rehabilitation | n/a | Yes | BACPR | No | Exercise |
| UK7 | Management of stable angina: A national clinical guideline, #96 [32] | 2007 | UK | Report | CGL | RCR | Yes | Scottish Intercollegiate Guidelines Network | n/a | Yes | SIGN/ NCG | No | General |
| UK8 | Joint British Societies’ consensus recommendations for the prevention of cardiovascular disease (JBS3) [33] | 2014 | UK | Journal | CS | PA + RCR | Yes | BCS/BHS/Diabetes UK/HEART UK/PCCS/The Stroke Association | Heart | Yes | PubMed | Yes | General |
| UK9 | Cardiac rehabilitation: A national clinical guideline, #57 [34] | 2002 | UK | Report | CGL | CRS | No | Scottish Intercollegiate Guidelines Network | n/a | Yes | SIGN/ NCG | No | Cardiac Rehabilitation |
| AUS1 | NHFA and CSANZ Australian clinical guidelines for the management of acute coronary syndromes 2016 [35–37] | 2016 | AUS | Journal | CGL | PA + RCR | Yes | National Heart Foundation of Australia; Cardiac Society of Australia and New Zealand | Heart, Lung and Circulation | Yes | CSANZ/ NHFA | Yes | General |
| AUS2 | National Heart Foundation of Australia physical activity recommendations for people with cardiovascular disease [38] | 2006 | AUS | Journal & Report | Recs | PA + RCR | No | National Heart Foundation of Australia | Medical Journal of Australia | Yes | NHFA | Yes | Exercise |
| AUS3 | ACRA core components of cardiovascular disease, secondary prevention and cardiac rehabilitation 2014 [39] | 2015 | AUS | Journal | CC | CRS | No | Australian Cardiovascular Health and Rehabilitation Association | Heart, Lung and Circulation | Yes | ACRA | Yes | Cardiac Rehabilitation |
| AUS4 | Recommended framework for cardiac rehabilitation ’04 [40] | 2004 | AUS | Report | Recs | CRS | Yes | Australian Cardiovascular Health and Rehabilitation Association; National Heart Foundation of Australia | n/a | Yes | ACRA/ NHFA | No | Cardiac Rehabilitation |
| AUS5 | Best practice guidelines for cardiac rehabilitation and secondary prevention [41] | 1999 | AUS | Report | CGL | CRS | No | Heart Research Centre; Human Services Victoria | n/a | Yes | ACRA | No | Cardiac Rehabilitation |
| AUS6 | A practitioner’s guide for cardiac rehabilitation [42] | 1999 | AUS | Report | Guide | CRS | No | Australian Cardiovascular Health and Rehabilitation Association | n/a | Yes | ACRA | No | Cardiac Rehabilitation |
| AUS7 | Reducing risk in heart disease: An expert guide to clinical practice for secondary prevention of coronary heart disease [43] | 2012 | AUS | Report | Guide | PA + RCR | Yes | National Heart Foundation of Australia; Cardiac Society of Australia and New Zealand | n/a | Yes | CSANZ/ NHFA/ NHMRC | No | General |
| NZ1 | Evidence-based best practice guideline: cardiac rehabilitation [44] | 2002 | NZ | Report | CGL | CRS | No | New Zealand Guidelines Group; National Heart Foundation New Zealand | n/a | Yes | NZGG/ NHFNZ | No | Cardiac Rehabilitation |
| NZ2 | Assessment and management of cardiovascular risk [45,46] | 2003/2013 | NZ | Report | CGL | PA + RCR | Yes | New Zealand Guidelines Group | n/a | Yes | NZGG/ NHFNZ | No | General |
| EUR1 | 2013 ESC guidelines on the management of stable coronary artery disease [47] | 2013 | EUR | Journal | CGL | PA + RCR | Yes | European Society of Cardiology | European Heart Journal | Yes | ESC | Yes | General |
| EUR2 | ESC guidelines for the management of acute myocardial infarction in patients presenting with ST-segment elevation [48] | 2012 | EUR | Journal | CGL | PA + RCR | Yes | European Society of Cardiology | European Heart Journal | Yes | ESC | Yes | General |
| EUR3 | 2016 European guidelines on cardiovascular disease prevention in clinical practice version [49] | 2016 | EUR | Journal | CGL | PA + RCR | Yes | European Society of Cardiology | European Heart Journal | Yes | ESC | Yes | General |
| EUR4 | Secondary prevention in the clinical management of patients with cardiovascular diseases. Core components, standards and outcome measures for referral and delivery [50] | 2012 | EUR | Journal | CC | CRS | No | European Association for Cardiovascular Prevention and Rehabilitation | European Journal of Preventive Cardiology | Yes | EACPR | Yes | Cardiac Rehabilitation |
| EUR5 | Secondary prevention through cardiac rehabilitation: from knowledge to implementation. [51] | 2010 | EUR | Journal | PS | CRS | No | European Association for Cardiovascular Prevention and Rehabilitation | European Journal of Preventive Cardiology | Yes | EACPR | Yes | Cardiac Rehabilitation |
| EUR6 | Secondary prevention through cardiac rehabilitation: physical activity counselling and exercise training [52] | 2010 | EUR | Journal | PS | CRS | No | European Association for Cardiovascular Prevention and Rehabilitation | European Journal of Preventive Cardiology | Yes | EACPR | Yes | Exercise |
| EUR7 | Secondary prevention through cardiac rehabilitation [53] | 2003 | EUR | Journal | PS | CRS | No | European Society of Cardiology | European Heart Journal | Yes | EACPR | Yes | Cardiac Rehabilitation |
| EUR8 | Physical activity for primary and secondary prevention [54] | 2003 | EUR | Journal | PS | PA + RCR | No | European Society of Cardiology | European Journal of Preventive Cardiology | Yes | ESC | Yes | Exercise |
| EUR9 | Clinical practice guidelines for physical therapy in cardiac rehabilitation [55,56] | 2013 | EUR | Journal | CGL | CRS | Yes | Royal Dutch Society for Physical Therapy | Netherland Heart Journal | Yes | G-I-N | Yes | Exercise |
| EUR10 | 2015 ESC guidelines for the management of acute coronary syndromes in patients presenting without persistent ST-segment elevation [57] | 2015 | EUR | Journal | CGL | PA + RCR | Yes | European Society of Cardiology | European Heart Journal | Yes | ESC | Yes | General |
| EUR11 | French Society of Cardiology guidelines for cardiac rehabilitation in adults [58] | 2012 | EUR | Journal | CGL | CRS | No | French Society of Cardiology | Archives of Cardiovascular Disease | Yes | PubMed | Yes | Cardiac Rehabilitation |
| EUR12 | Recommendations for resistance exercise in cardiac rehabilitation [59] | 2004 | EUR | Journal | Recs | CRS | No | European Society of Cardiology | European Journal of Preventive Cardiology | No | EACPR | Yes | Exercise |

AACVPR, American Association of Cardiovascular and Pulmonary Rehabilitation; AATS, American Association for Thoracic Surgery; ACCF, American College of Cardiology Foundation; ACP, American College of Physicians; ACRA, Australian Cardiovascular Health and Rehabilitation Association; ACSM, American College of Sports Medicine; AHA, American Heart Association; AUS, Australia; BACPR, British Association for Cardiovascular Prevention and Rehabilitation; BCS, British Cardiac Society; BHS, British Hypertension Society; CACPR, Canadian Association of Cardiovascular Prevention and Rehabilitation; CAN, Canada; CC, Core Components; CCVS, Canadian Cardiovascular Society; CGL, Clinical Guideline; CMA, Canadian Medical Association; CRS, Cardiac Rehabilitation specific; CS, Consensus Conference/Statement; CSANZ, Cardiac Society of Australia and New Zealand; EACPR, European Association for Cardiovascular Prevention and Rehabilitation; ESC, European Society of Cardiology; EUR, Europe; G-I-N, Guidelines International Network; NCG, National Guideline Clearinghouse; NHFA, National Heart Foundation of Australia; NHFNZ, National Heart Foundation of New Zealand; NHMRC, National Health and Medical Research Council; NICE, National Institute for Health and Care Excellence; NZ, New Zealand; NZGG, New Zealand Guidelines Group; PAA, Physical Activity advice; PA + RCR, Physical Activity advice and refer to Cardiac Rehabilitation; PCCS, Primary Care Cardiovascular Society; PCNA, Preventive Cardiovascular Nurses Association; PS, Position Stand; RCR, Refer to Cardiac Rehabilitation; Recs, Recommendations; SCAI, Society for Cardiovascular Angiography and Interventions; SIGN, Scottish Intercollegiate Guidelines Network; SS, Scientific Statement; ST, Standards; STS, Society of Thoracic Surgeons; UK, United Kingdom; USA, United States of America; * an executive summary of the publication is indexed by PubMed

**Table Reference List**

1. University of Michigan Health System. Secondary prevention of ischemic heart disease and stroke in adults. Ann Arbor (MI): University of Michigan Health System; 2014.

2. American College of Sports Medicine. Chapter 9: Exercise Prescription for Patients with Cardiovascular and Cerebrovascular Disease. ACSM’s Guidel. Exerc. Test. Prescr. 9th ed. Philadelphia: Lippincott Williams and Wilkins; 2013.

3. Fleg JL, Forman DE, Berra K, Bittner V, Blumenthal JA, Chen MA, et al. Secondary prevention of atherosclerotic cardiovascular disease in older adults: A scientific statement from the American heart association. Circulation. 2013;128:2422–46.

4. Institute for Clinical Systems Improvement: Goblirsch G, Bershow S, Cummings K, Hayes R, Kokoszka M, Lu Y, et al. Stable Coronary Artery Disease. Institute for Clinical Systems Improvement; 2013.

5. American Association of Cardiovascular and Pulmonary Rehabilitation. Guidelines for Cardiac Rehabilitation and Secondary Prevention Programs. 5th ed. Champaign, IL: Human Kinetics; 2013.

6. Fihn SD, Gardin JM, Abrams J, Berra K, Blankenship JC, Dallas AP, et al. 2012 ACCF / AHA / ACP / AATS / PCNA / SCAI / STS Guideline for the Diagnosis and Management of Patients With Stable Ischemic Heart Disease. J. Am. Coll. Cardiol. 2012;60.

7. Fihn SD, Blankenship JC, Alexander KP, Bittl JA, Byrne JG, Fletcher BJ, et al. 2014 ACC/AHA/AATS/PCNA/SCAI/STS Focused Update of the Guideline for the Diagnosis and Management of Patients With Stable Ischemic Heart Disease: A Report of the American College of Cardiology/American Heart Association Task Force on Practice Guidelines, a. Circulation. 2014;130:1749–67.

8. Smith SC, Benjamin EJ, Bonow RO, Braun LT, Creager MA, Franklin BA, et al. AHA/ACCF secondary prevention and risk reduction therapy for patients with coronary and other atherosclerotic vascular disease: 2011 update. Circulation. 2011;124:2458–73.

9. Institute for Clinical Systems Improvement. Health Care Guideline: Diagnosis and Treatment of Chest Pain and Acute Coronary Syndrome ( ACS ). Institute for Clinical Systems Improvement; 2012.

10. Levine GN, Bates ER, Blankenship JC, Bailey SR, Bittl JA, Cercek B, et al. 2011 ACCF/AHA/SCAI Guideline for Percutaneous Coronary Intervention: a report of the American College of Cardiology Foundation/American Heart Association Task Force on Practice Guidelines and the Society for Cardiovascular Angiography and Interventions. Circulation. United States; 2011;124:e574-651.

11. Levine GN, Bates ER, Blankenship JC, Bailey SR, Bittl JA, Cercek B, et al. 2015 ACC/AHA/SCAI Focused Update on Primary Percutaneous Coronary Intervention for Patients With ST-Elevation Myocardial Infarction. J. Am. Coll. Cardiol. 2016;67:1235–50.

12. O’Gara PT, Kushner FG, Ascheim DD, Casey DEJ, Chung MK, de Lemos JA, et al. 2013 ACCF/AHA guideline for the management of ST-elevation myocardial infarction: a report of the American College of Cardiology Foundation/American Heart Association Task Force on Practice Guidelines. Circulation. United States; 2013;127:e362-425.

13. Fraker TD, Fihn SD, Gibbons RJ, Abrams J, Chatterjee K, Daley J, et al. 2007 Chronic Angina Focused Update of the ACC/AHA 2002 Guidelines for the Management of Patients With Chronic Stable Angina. Circulation. 2007;116:2762–72.

14. Balady GJ, Williams M, Ades P, Bittner V, Comoss P, Foody JM, et al. Core components of cardiac rehabilitation/secondary prevention programs: 2007 update. Circulation. 2007;115:2675–82.

15. Fletcher GF, Ades P a., Kligfield P, Arena R, Balady GJ, Bittner V a., et al. Exercise standards for testing and training: A scientific statement from the American Heart Association. Circulation. 2013;128:873–934.

16. American College of Sports Medicine. American College of Sports Medicine Position Stand: Exercise for Patients with Coronary Artery Disease. Med. Sci. Sports Exerc. 1994;26:i–v.

17. Williams MA, Haskell WL, Ades PA, Amsterdam EA, Bittner V, Franklin BA, et al. Resistance exercise in individuals with and without cardiovascular disease: 2007 update: A scientific statement from the American Heart Association Council on Clinical Cardiology and Council on Nutrition, Physical Activity, and Metabolism. Circulation. 2007;116:572–84.

18. Leon AS, Franklin BA, Costa F, Balady GJ, Berra KA, Stewart KJ, et al. Cardiac Rehabilitation and Secondary Prevention of Coronary Heart Disease. Circulation. 2005;111:369–76.

19. Thompson PD, Buchner D, Piña IL, Balady GJ, Williams M a., Marcus BH, et al. Exercise and physical activity in the prevention and treatment of atherosclerotic cardiovascular disease. Circulation. 2003;107:3109–16.

20. Mosca L, Benjamin EJ, Berra K, Bezanson JL, Dolor RJ, Lloyd-Jones DM, et al. Effectiveness-based guidelines for the prevention of cardiovascular disease in women-2011 update. Circulation. 2011;123:1243–62.

21. Kulik A, Ruel M, Jneid H, Ferguson TB, Hiratzka LF, Ikonomidis JS, et al. Secondary prevention after coronary artery bypass graft surgery: a scientific statement from the American Heart Association. Circulation. 2015;131:1–39.

22. Amsterdam EA, Wenger NK, Brindis RG, Casey DE, Ganiats TG, Holmes DR, et al. 2014 AHA/ACC Guideline for the Management of Patients With Non-ST-Elevation Acute Coronary Syndromes: A Report of the American College of Cardiology/American Heart Association Task Force on Practice Guidelines. Circulation. 2014;130:e344–426.

23. Canadian Association of Cardiovascular Prevention and Rehabilitation. Canadian guidelines for cardiac rehabilitation and cardiovascular disease prevention: Translating knowledge into action. 3rd ed. Winnipeg, Man: Canadian Association of Cardiovascular Prevention and Rehabilitation; 2009.

24. Kalina PRB, Leblanc VA, Hajigeorgiou M, Cason N, Gillespie C, Rabczak M, et al. Canadian Cardiovascular Society 2000 Consensus Conference: Women and Ischemic Heart Disease. Can. J. Cardiol. 2001;17:Supplement D.

25. Mancini GBJ, Gosselin G, Chow B, Kostuk W, Stone J, Yvorchuk KJ, et al. Canadian Cardiovascular Society Guidelines for the Diagnosis and Management of Stable Ischemic Heart Disease. Can. J. Cardiol. Canadian Cardiovascular Society; 2014;30:837–49.

26. Canadian Cardiovascular Society. Canadian Cardiovascular Society Consensus Conference 2002: Management of Heart Disease in the Elderly Patient. Canadian Cardiovascular Society; 2002.

27. National Institute for Health and Care Excellence. MI – secondary prevention: Secondary prevention in primary and secondary care for patients following a myocardial infarction. NICE guideline (CG172). National Institute for Health and Care Excellence; 2013.

28. British Association for Cardiovascular Prevention and Rehabilitation. The BACPR standards and core components for cardiovascular disease prevention and rehabilitation 2012 (2nd edition). London: The British Association for Cardiovascular Prevention and Rehabilitation; 2012.

29. National Institute for Health and Clinical Excellence. Management of stable angina. NICE guideline (CG126). National Institute for Health and Care Excellence; 2012.

30. National Institute for Health and Clinical Excellence. Unstable Angina and NSTEMI: The Early Management of Unstable Angina and Non-ST-Segment-Elevation Myocardial Infarction. NICE guideline (CG94). National Institute for Health and Clinical Excellence; 2010.

31. Association of Chartered Physiotherapists in Cardiac Rehabilitation. ACPICR standards for physical activity and exercise in the cardiac population 2015. Association of Chartered Physiotherapists in Cardiac Rehabilitation; 2015.

32. Scottish Intercollegiate Guidelines Network (SIGN). Management of stable angina. (SIGN publication no. 96). Edinburgh: SIGN; 2007.

33. British Cardiac Society, British Hypertension Society, Diabetes UK, HEART UK, Primary Care Cardiovascular Society, The Stroke Association. Joint British Societies’ consensus recommendations for the prevention of cardiovascular disease (JBS3). Heart. 2014;100:ii1-ii67.

34. Scottish Intercollegiate Guidelines Network (SIGN). Cardiac Rehabilitation. (SIGN publication no. 57). Edinburgh: SIGN; 2002.

35. Acute Coronary Syndrome Guidelines Working Group. Guidelines for the management of acute coronary syndromes 2006. Med. J. Aust. 2006;184:S9–29.

36. Chew DP, Aroney CN, Aylward PE, Kelly A-M, White HD, Tideman PA, et al. 2011 Addendum to the National Heart Foundation of Australia/Cardiac Society of Australia and New Zealand Guidelines for the management of acute coronary syndromes (ACS) 2006. Heart. Lung Circ. Australia; 2011;20:487–502.

37. Chew DP, Scott IA, Cullen L, French J, Briffa TG, Tideman PA, et al. National Heart Foundation of Australia & Cardiac Society of Australia and New Zealand: Australian Clinical Guidelines for the Management of Acute Coronary Syndromes 2016. Hear. Lung Circ. 2016;25:895–951.

38. Briffa TG, Allan R, Maiorana A, Oldenburg B, Sammel N, Stubbs A, et al. National Heart Foundation of Australia physical activity recommendations for people with cardiovascular disease. Sydney: National Heart Foundation of Australia; 2006.

39. Woodruffe S, Neubeck L, Clark R, Gray K, Ferry C, Finan J, et al. Australian Cardiovascular Health and Rehabilitation Association (ACRA) core components of cardiovascular disease secondary prevention and cardiac rehabilitation 2014. Hear. Lung Circ. Australian and New Zealand Society of Cardiac and Thoracic Surgeons (ANZSCTS) and the Cardiac Society of Australia and New Zealand (CSANZ); 2015;24:430–41.

40. Australian Cardiovascular Health and Rehabilitation Association & National Heart Foundation. Recommended framework for cardiac rehabilitation ’04. Australian Cardiovascular Health and Rehabilitation Association National Heart Foundation; 2004.

41. Goble AJJ, Worcester MUC. Best Practice Guidelines for Cardiac Rehabilitation and Secondary Prevention. Carlton, VIC: Human Services Victoria; 1999.

42. Australian Cardiovascular Health and Rehabilitation Association. A Practitioner’s Guide for Cardiac Rehabilitation 1999. Australian Cardiovascular Health and Rehabilitation Association; 1999.

43. National Heart Foundation of Australia & Cardiac Society of Australia and New Zealand. Reducing risk in heart disease: An expert guide to clinical practice for secondary prevention of coronary heart disease. Melbourne: National Heart Foundation of Australia; 2012.

44. New Zealand Guidelines Group, Heart Foundation. Evidence-Based Best Practice Guideline: Cardiac Rehabilitation. Wellington, NZ: New Zealand Guidelines Group; 2002.

45. New Zealand Guidelines Group. Assessment and management of cardiovascular risk. Wellington, NZ; 2003.

46. New Zealand Guidelines Group. Cardiovascular Disease Risk Assessment. New Zeal. Prim. Care Handb. 2012. Wellington, NZ; 2013.

47. Montalescot G, Sechtem U, Achenbach S, Andreotti F, Arden C, Budaj A, et al. 2013 ESC guidelines on the management of stable coronary artery disease: the Task Force on the management of stable coronary artery disease of the European Society of Cardiology. Eur. Heart J. 2013;34:2949–3003.

48. Steg PG, James SK, Atar D, Badano LP, Lundqvist CB, Borger M a., et al. ESC Guidelines for the management of acute myocardial infarction in patients presenting with ST-segment elevation. Eur. Heart J. 2012;33:2569–619.

49. Piepoli MF, Hoes AW, Agewall S, Albus C, Brotons C, Catapano AL, et al. 2016 European Guidelines on cardiovascular disease prevention in clinical practice. Eur. Heart J. 2016;37:2315–81.

50. Piepoli MF, Corra U, Adamopoulos S, Benzer W, Bjarnason-Wehrens B, Cupples M, et al. Secondary prevention in the clinical management of patients with cardiovascular diseases. Core components, standards and outcome measures for referral and delivery. Eur. J. Prev. Cardiol. 2012;Epub Jun 2:1–18.

51. Piepoli MF, Corrà U, Benzer W, Bjarnason-Wehrens B, Dendale P, Gaita D, et al. Secondary prevention through cardiac rehabilitation: from knowledge to implementation. A position paper from the Cardiac Rehabilitation Section of the European Association of Cardiovascular Prevention and Rehabilitation. Eur. J. Cardiovasc. Prev. Rehabil. 2010;17:1–17.

52. Corr U, Carré F, Heuschmann P, Hoffmann U, Verschuren M, Halcox J, et al. Secondary prevention through cardiac rehabilitation: Physical activity counselling and exercise training. Eur. Heart J. 2010;31.

53. Bassand J-P, Hamm CW, Ardissino D, Boersma E, Budaj A, Fernández-Avilés F, et al. Guidelines for the diagnosis and treatment of non-ST-segment elevation acute coronary syndromes. Eur. Heart J. 2007;28:1598–660.

54. Giannuzzi P, Mezzani A, Saner H, Björnstad H, Fioretti P, Mendes M, et al. Physical activity for primary and secondary prevention. Position paper of the Working Group on Cardiac Rehabilitation and Exercise Physiology of the European Society of Cardiology. Eur. J. Cardiovasc. Prev. Rehabil. 2003;10:319–27.

55. Achttien RJ, Staal JB, van der Voort S, Kemps HMC, Koers H, Jongert MWA, et al. Exercise-based cardiac rehabilitation in patients with coronary heart disease: A practice guideline. Netherlands Hear. J. 2013;21:429–38.

56. Royal Dutch Society for Physical Therapy. KNGF Clinical Practice Guideline for physical therapy in patients undergoing cardiac rehabilitation. Dutch J. Phys. Ther. 2011;121:Supp.

57. Roffi M, Patrono C, Collet J-P, Mueller C, Valgimigli M, Andreotti F, et al. 2015 ESC Guidelines for the management of acute coronary syndromes in patients presenting without persistent ST-segment elevation. Eur. Heart J. Eur Soc Cardiology; 2015;ehv320.

58. Pavy B, Iliou MC, Vergès-Patois B, Brion R, Monpère C, Carré F, et al. French Society of Cardiology guidelines for cardiac rehabilitation in adults. Arch. Cardiovasc. Dis. 2012;105:309–28.

59. Bjarnason-Wehrens B, Mayer-Berger W, Meister ER, Baum K, Hambrecht R, Gielen S. Recommendations for resistance exercise in cardiac rehabilitation. Recommendations of the German Federation for Cardiovascular Prevention and Rehabilitation. Eur. J. Cardiovasc. Prev. Rehabil. 2004;11:352–61.
