# Supplementary material for: Exploration of the methodological quality and clinical usefulness of a cross-sectional sample of published guidance about exercise training and physical activity for the secondary prevention of coronary heart disease
Source: BMC Cardiovasc Disord. 2017 Jun 13;17:153. doi: 10.1186/s12872-017-0589-z (PMC5470313; doi:10.1186/s12872-017-0589-z)
Supplement: Supplementary file 4 — Table. AGREE II domain scores (%) for all included publications. (DOCX 27 kb) [file 12872_2017_589_MOESM4_ESM.docx]

**Additional file 4.** AGREE II domain scores (%) for all included publications

| **I.D** | **Publication Title** | **Guidance Type** | **AGREE II Domain (%)** | | | | | |
| --- | --- | --- | --- | --- | --- | --- | --- | --- |
|  |  |  | ***Scope and Purpose*** | ***Stakeholder Involvement*** | ***Rigour of Development*** | ***Clarity of Presentation*** | ***Applicability*** | ***Editorial Independence*** |
| USA1 | Secondary prevention of ischemic heart disease and stroke in adults | CGL | 53 | 33 | 37 | 75 | 13 | 46 |
| USA2 | ACSM's guidelines for exercise testing and prescription (9th edition). Chapter 9: Exercise prescription for patients with cardiovascular and cerebrovascular disease | CGL* | 69 | 47 | 40 | 97 | 35 | 8 |
| USA3 | Secondary prevention of atherosclerotic cardiovascular disease in older adults | Other | 61 | 22 | 32 | 56 | 31 | 67 |
| USA4 | Stable coronary artery disease | CGL | 83 | 67 | 68 | 72 | 56 | 96 |
| USA5 | Guidelines for cardiac rehabilitation and secondary prevention programs, 5th edition | CGL* | 53 | 42 | 39 | 89 | 75 | 0 |
| USA6 | Guideline for the diagnosis and management of patients with stable ischemic heart disease | CGL | 78 | 50 | 71 | 83 | 27 | 92 |
| USA7 | AHA/ACCF secondary prevention and risk reduction therapy for patients with coronary and other atherosclerotic vascular disease: 2011 update | CGL | 42 | 36 | 66 | 83 | 21 | 88 |
| USA8 | Diagnosis and treatment of chest pain and acute coronary syndrome | CGL | 72 | 61 | 64 | 89 | 69 | 88 |
| USA9 | Guideline for percutaneous coronary intervention | CGL | 83 | 53 | 78 | 75 | 48 | 92 |
| USA10 | Guideline for the management of ST-elevation myocardial infarction | CGL | 56 | 53 | 72 | 81 | 31 | 92 |
| USA11 | 2007 Chronic angina focused update of the 2002 guidelines for the management of patients with chronic stable angina | CGL | 25 | 33 | 46 | 72 | 21 | 88 |
| USA12 | Core components of cardiac rehabilitation/secondary prevention programs: 2007 update | Other | 56 | 47 | 21 | 86 | 23 | 79 |
| USA13 | Exercise standards for testing and training | Other | 58 | 44 | 32 | 50 | 23 | 88 |
| USA14 | Exercise for patients with coronary artery disease | Other | 50 | 8 | 19 | 69 | 21 | 13 |
| USA15 | Resistance exercise in individuals with and without cardiovascular disease: 2007 update | Other | 67 | 31 | 28 | 75 | 15 | 75 |
| USA16 | Cardiac rehabilitation and secondary prevention of coronary heart disease | Other | 72 | 17 | 23 | 42 | 23 | 79 |
| USA17 | Exercise and physical activity in the prevention and treatment of atherosclerotic cardiovascular disease | Other | 53 | 25 | 29 | 67 | 15 | 79 |
| USA18 | Effectiveness-based guidelines for the prevention of cardiovascular disease in women—2011 update | CGL | 47 | 47 | 53 | 58 | 46 | 88 |
| USA19 | Secondary prevention after coronary artery bypass graft surgery | Other | 50 | 31 | 55 | 64 | 25 | 88 |
| USA20 | 2014 AHA/ACC guideline for the management of patients with non–ST-elevation acute coronary syndromes | CGL | 61 | 44 | 75 | 72 | 31 | 100 |
| CAN1 | Canadian guidelines for cardiac rehabilitation and cardiovascular disease prevention: Translating knowledge into action, 3rd edition | CGL | 81 | 58 | 70 | 97 | 77 | 92 |
| CAN2 | Canadian Cardiovascular Society 2000 consensus conference: Women and ischemic heart disease | Other | 61 | 44 | 41 | 53 | 21 | 75 |
| CAN3 | Canadian Cardiovascular Society guidelines for the diagnosis and management of stable ischemic heart disease | CGL | 58 | 42 | 44 | 64 | 10 | 92 |
| CAN4 | Management of heart disease in the elderly patient | Other | 61 | 33 | 42 | 78 | 35 | 71 |
| UK1 | Cardiovascular disease: risk assessment and reduction, including lipid modification, #181 | CGL | 100 | 78 | 92 | 78 | 79 | 75 |
| UK2 | MI – secondary prevention: Secondary prevention in primary and secondary care for patients following a myocardial infarction, #172 | CGL | 89 | 92 | 82 | 86 | 71 | 92 |
| UK3 | BACPR standards and core components for cardiovascular disease prevention and rehabilitation 2012 | Other | 78 | 64 | 33 | 75 | 67 | 58 |
| UK4 | Management of stable angina, #126 | CGL | 94 | 78 | 90 | 81 | 75 | 83 |
| UK5 | Unstable angina and NSTEMI: The early management of unstable angina and non-ST-segment-elevation myocardial infarction, #94 | CGL | 92 | 86 | 83 | 78 | 75 | 88 |
| UK6 | ACPICR Standards for physical activity and exercise in the cardiac population 2015 | Other | 86 | 44 | 31 | 81 | 38 | 4 |
| UK7 | Management of stable angina: A national clinical guideline, #96 | CGL | 67 | 69 | 83 | 75 | 54 | 88 |
| UK8 | Joint British Societies’ consensus recommendations for the prevention of cardiovascular disease (JBS3) | Other | 72 | 56 | 35 | 72 | 44 | 83 |
| UK9 | Cardiac Rehabilitation: A national clinical guideline, #57 | CGL | 61 | 75 | 75 | 86 | 56 | 67 |
| AUS1 | Australian clinical guidelines for the management of acute coronary syndromes 2016 | CGL | 50 | 67 | 35 | 78 | 52 | 75 |
| AUS2 | National Heart Foundation of Australia physical activity recommendations for people with cardiovascular disease | Other | 67 | 64 | 55 | 78 | 27 | 58 |
| AUS3 | ACRA core components of cardiovascular disease secondary prevention and cardiac rehabilitation 2014 | Other | 75 | 44 | 25 | 75 | 44 | 21 |
| AUS4 | Recommended framework for cardiac rehabilitation ’04 | Other | 75 | 42 | 26 | 64 | 46 | 38 |
| AUS5 | Best practice guidelines for cardiac rehabilitation and secondary prevention | CGL | 75 | 82 | 57 | 86 | 73 | 29 |
| AUS6 | A practitioner’s guide for cardiac rehabilitation | Other | 58 | 17 | 13 | 61 | 52 | 0 |
| AUS7 | Reducing risk in heart disease: An expert guide to clinical practice for secondary prevention of coronary heart disease | Other | 50 | 33 | 38 | 69 | 33 | 79 |
| NZ1 | Evidence-based best practice guideline: cardiac rehabilitation | CGL | 97 | 83 | 78 | 89 | 77 | 79 |
| NZ2 | Assessment and management of cardiovascular risk | CGL | 72 | 67 | 60 | 83 | 67 | 67 |
| EUR1 | 2013 ESC guidelines on the management of stable coronary artery disease | CGL | 64 | 42 | 71 | 64 | 56 | 96 |
| EUR2 | ESC guidelines for the management of acute myocardial infarction in patients presenting with ST-segment elevation | CGL | 58 | 39 | 67 | 61 | 56 | 88 |
| EUR3 | 2016 European guidelines on cardiovascular disease prevention in clinical practice version | CGL | 72 | 47 | 70 | 78 | 73 | 76 |
| EUR4 | Secondary prevention in the clinical management of patients with cardiovascular diseases. Core components, standards and outcome measures for referral and delivery | Other | 69 | 44 | 12 | 75 | 75 | 96 |
| EUR5 | Secondary prevention through cardiac rehabilitation: from knowledge to implementation. | Other | 64 | 33 | 27 | 92 | 40 | 4 |
| EUR6 | Secondary prevention through cardiac rehabilitation: physical activity counselling and exercise training | Other | 44 | 31 | 28 | 86 | 31 | 46 |
| EUR7 | Secondary prevention through cardiac rehabilitation | Other | 64 | 44 | 19 | 61 | 33 | 0 |
| EUR8 | Physical activity for primary and secondary prevention | Other | 64 | 31 | 26 | 61 | 17 | 0 |
| EUR9 | Clinical practice guidelines for physical therapy in cardiac rehabilitation | CGL | 94 | 72 | 54 | 81 | 46 | 29 |
| EUR10 | 2015 ESC guidelines for the management of acute coronary syndromes in patients presenting without persistent ST-segment elevation | CGL | 47 | 36 | 67 | 69 | 60 | 96 |
| EUR11 | French Society of Cardiology guidelines for cardiac rehabilitation in adults | CGL | 39 | 19 | 32 | 78 | 40 | 29 |
| EUR12 | Recommendations for resistance exercise in cardiac rehabilitation | Other | 53 | 22 | 22 | 69 | 10 | 8 |

AUS, Australia; CAN, Canada; CGL, Clinical Guideline; EUR, Europe; NZ, New Zealand; Other, other type of guidance document; UK, United Kingdom; USA, United States of America; * classified as a guideline based on self-reported title but would fail to meet the IOM definition of a clinical practice guideline
